# Perceptions and Experiences of Inequity for Women of Refugee Background Having a Baby during the COVID-19 Pandemic in Melbourne, Australia

**DOI:** 10.3390/ijerph21040481

**Published:** 2024-04-15

**Authors:** Fran Hearn, Stephanie J. Brown, Josef Szwarc, Shadow Toke, May Alqas Alias, Maryaan Essa, Shogoufa Hydari, Ashay Baget, Elisha Riggs

**Affiliations:** 1 Intergenerational Health, Murdoch Children’s Research Institute, Parkville, VIC 3052, Australia; 2 Department of General Practice, University of Melbourne, Parkville, VIC 3052, Australia; 3 Department of Paediatrics, University of Melbourne, Parkville, VIC 3052, Australia; 4 South Australian Health and Medical Research Institute, Adelaide, SA 5001, Australia; 5 The Victorian Foundation for Survivors of Torture Inc., Brunswick, VIC 3056, Australia

**Keywords:** refugee, maternal health, health equity, COVID-19, qualitative research

## Abstract

Listening to What Matters is an exploratory descriptive qualitative study that aimed to (1) understand how women of refugee background in Melbourne, Australia experienced access to health information and maternity and/or early parenting care during the COVID-19 pandemic and (2) whether pandemic health directives had an impact on structural inequities for women of refugee background who received maternity and/or early parenting care during the COVID-19 pandemic. Semi-structured interviews were conducted with 41 participants including 17 women of refugee background, who identified as belonging to the Karen, Assyrian Chaldean, Iraqi, Syrian, Afghan, Sudanese, or South Sudanese communities and 24 health and social care professionals who identified as providing pregnancy or early parenting care during the pandemic in the north western suburbs of Melbourne. Interviews with women were conducted in preferred languages by community researchers. Interviews with professionals were conducted in English by researchers. Reflexive thematic data analysis included constructivist positionality and a trauma and violence informed approach. The results reported in this paper include three themes, with four accompanying subthemes, as follows: theme (1), ‘Structural inequities and the toll of the pandemic’; theme (2), ‘Supportive infrastructure’; and theme (3), ‘Cultural safety during the pandemic’. The results demonstrate that cumulative negative impacts such as unequal access to health information, family separation and isolation, inadequate household income, and mental and social health concerns had the potential to amplify pre-existing structural inequities for women of refugee background. Community engagement facilitated by bicultural workers, interpreters, and trusted care providers facilitated fast-paced, two-way communication that built capacity and health literacy for women who were unable to speak English and unfamiliar with the health care system and, improved experiences of care. More research is needed to understand how the intersectional cumulative impacts of structural inequities have affected maternal and neonatal health outcomes for women of refugee background during the pandemic, as well as any differences in maternal and neonatal health outcomes between Australian-born and refugee background women and babies.

## 1. Introduction

### 1.1. Health Equity for Women of Refugee Background

In Australia and other high-income countries, women of refugee background having a baby continue to experience inequitable maternal and perinatal health outcomes, compared to women born in high-income countries [1,2,3,4,5,6]. Women of refugee background are more likely to experience difficulty understanding health information [4], receive fewer than recommended antenatal appointments [1], give birth to a low birth weight or stillborn baby [2,5,6], and experience mental or social health concerns [2]. It is important to note that these comparisons do not include First Nations [7], LGBTQIA+ [8], and other [9] women, individuals, families, and communities that are more likely to experience impacts to their physical, mental, and/or social health, due to the intersection of systemic factors such as discrimination and marginalisation. Equity-oriented change has been identified as a global health priority, to meet the needs of women of refugee background having a baby—including improving the understanding of and access to culturally safe maternity (pregnancy, labour, birth, and/or postnatal) care [3,10,11]. Health equity during the perinatal period means that women, people, families, and communities of refugee background have a *“fair chance to reach their full health potential and are not disadvantaged by social, economic and environmental conditions”* [12]. For example, by being able to live in a safe and financially secure environment and by being able to access health information and health care in their own language when needed [13,14]. 

In this paper, we use the language ‘women of refugee background’ inclusively to refer to all women with refugee or refugee-like experiences of forced displacement, persecution, and/or human rights violations, regardless of migration visa category, including those seeking asylum [15]. Australia’s migration and visa allocation system is complex and offers temporary and permanent settlement for migrants and refugees through different programs, depending on whether a person is seeking education, employment, or protection [16,17]. Depending on the type of visa a person has, they may or may not have the right to work, access universal health care [Medicare], or welfare such as social security payments and services [Services Australia] in Australia [16,17,18,19]. For example, a visa that recognises refugee status typically includes access to these rights, while a visa that acknowledges an application for asylum may not include access to these rights [1,2,20,21,22]. This means there are women of refugee background living in Australia who, due to their visa type, experience significant poverty and limited access to health care [1,2,20,21,22]. In the State of Victoria, where this study is based, the State Government does provide free access to public hospitals and some community health services, including the Maternal Child Health Service (which tracks child growth and development over the first five years of life) for those not eligible for Medicare [23]. Otherwise, community-based humanitarian, charitable, religious, non-denominational, non-government, or not- for-profit organisations, and refugee and migrant specific health and social care services, are the main sources of assistance [21]. 

### 1.2. The COVID-19 Pandemic in Melbourne, Australia in 2020

The SARS-CoV-2 [COVID-19] pandemic was declared by the World Health Organization on 11 March 2020 [24]. At the time this study was conducted, Australia had experienced two significant ‘waves’ of infection [25]. The first wave was predominantly associated with international travel and was suppressed via a nationwide lockdown during March to May of 2020 [25,26]. The second wave was unique to the State of Victoria and was driven by community transmission [26,27,28]. Policy and practice changes that eliminated community transmission and prevented the overwhelm of the health care system included a second lockdown. This occurred throughout July to October of 2020, and is now considered one of the most stringent and lengthy lockdowns in the world [26,27,28]. While Australia’s public health strategy has evolved over time, from elimination to tolerance of community transmission and ‘living with’ COVID-19 [26], fieldwork for this study occurred during August and September of 2020—in the midst of the second Victorian lockdown, prior to the development of COVID-19 vaccines and prior to the emergence of later COVID-19 ‘variants of concern’ that influenced the course of the pandemic [29] and subsequent public health directives. Key conditions or events experienced during the second Victorian lockdown that are relevant to the results reported in this paper are outlined, below, in Table 1.

### 1.3. Having a Baby during the Pandemic

The unprecedented nature of the pandemic and rapid pace of the State Government’s public health response necessitated massive changes to the health care system, implemented in a short period of time [25,31,32,33,34,35]. In practice, the application of health advice was often inconsistent and varied between organisations [35,41]. Generally, modifications to maternity and early parenting care aimed to reduce viral transmission by minimising face-to-face encounters and by wearing masks and other PPE. Some face-to-face appointments were shortened, telehealth and telephone-based care were widely adopted, and the presence of partners and support people was limited during antenatal and postnatal appointments, as well as during labour and birth [25,31,32,33,34,35,42]. Research has since established that women having a baby during this period of the pandemic experienced significant negative impacts to their social, emotional, and mental health, including an increase in depression and anxiety [43], as well as distress, loneliness, and isolation [35,42,44,45]. For women of refugee background, experiences of having a baby during the pandemic were further impacted by the intersection of pandemic conditions with factors associated with forced migration and settlement in a new country [46]. These factors potentially include but are not limited to low health and system literacy, limited access to health information in their preferred language/s, limited access to health care, poverty, discrimination, prior experiences of trauma, and indefinite separation from friends, families, and community networks overseas [47,48,49,50]. 

### 1.4. Research Aims

Research that examines the experiences of women having a baby during the pandemic continues to emerge [35,42,43,44,45]. However, little has been published specifically addressing the experiences of refugee background women [46,49,51]. To the best of our knowledge, no other peer reviewed research has been published regarding the experiences of refugee background women having a baby during the pandemic in Victoria, Australia—meaning that the Listening to What Matters study addresses an important gap in the literature. Drawing on interviews with women of refugee background living in the north western suburbs of Melbourne, Australia and health and social care professionals providing care to families living in this part of Melbourne, this paper aims to achieve the following:

- Understand how women and families of refugee background experienced access to health information and maternity and/or early parenting care during the COVID-19 pandemic.
- Understand whether pandemic health directives had an impact on structural inequities for women and families of refugee background who received maternity and/or early parenting care during the COVID-19 pandemic. ‘Structural inequity’ refers to the interplay of systemic determinants known to inequitably affect health and wellbeing, including geographic, economic, social, and cultural factors. For example, where and how people live and work [52,53,54].

## 2. Materials and Methods

### 2.1. Study Design

Listening to What Matters is an exploratory descriptive qualitative study [55] that is a collaboration between the Stronger Futures Centre of Research Excellence [56] and The Victorian Foundation for Survivors of Torture [Foundation House], an agency providing counselling and other services to people of refugee background, including those who have had traumatic resettlement experiences [57]. Constructivist methodology [58], a trauma and violence informed [52,53,59,60] approach, and reflexive thematic analysis [61] were used to write this paper. Constructivism and reflexive thematic analysis acknowledge the impact of our language, culture, and experiences on our understanding of reality [58,61,62]. The main benefit of conducting a reflexive thematic analysis was that data could be analysed with the aims of this paper in mind—with a view to being able to make policy and practice recommendations that address health equity considerations for women and families of refugee background. This study has generated two prior outputs on the lived experiences of women [63] and midwives [41] during the pandemic that used interpretive phenomenological analysis [64].

### 2.2. Eligibility, Participant Recruitment, and Consent

This study was conducted with (1) women of refugee background who received maternity and/or early parenting care during the pandemic in the north western regions of Melbourne and who identify as belonging to the Karen, Assyrian Chaldean, Iraqi, Syrian, Afghan, Sudanese, or South Sudanese communities and (2) health and social care professionals who identify as providing maternity and/or early parenting care during the pandemic in the north western regions of Melbourne. Geographic eligibility was determined by the scope of the funding organisation—the North Western Melbourne Primary Health Network [NWMPHN] [20]. Relevant local council areas included Hume, Moreland, Darebin, Yarra, Melbourne, Maribyrnong, Hobsons Bay, Brimbank, Moonee Valley, Melton, Wyndham, Moorabool, and the Macedon Ranges. A diverse range of families access maternity and early parenting services in these local areas, which include a high number of people of refugee background compared to other areas in Victoria [20,65].

Eligible participants able to provide meaningful data were strategically recruited using purposive and snowball sampling [66]. Women were recruited by community researchers [ST, MAA, ME, SH, AB] [15,67] through social, community and professional networks, as well as through social media such as WhatsApp, Viber, and Facebook. Community researchers were culturally and linguistically matched to the background of prospective participants and facilitated the meaningful inclusion of women from some of the largest communities of refugee background resettled in the north western suburbs of Victoria, Australia (where this study was based) [20,65]. Each community researcher tailored their recruitment approach to suit the needs and preferences of their community. Digital flyers and videos inviting participants to the study were created in community languages and included a Research Electronic Data Capture [REDCap] [68] hyperlink to translated participant information. Community researchers that could speak the participant’s preferred language scheduled each interview, answered questions about the study, and obtained informed e-consent. Health and social care professionals were recruited by researchers [FH, ER] through the Refugee and Migrant Health Research Program’s professional networks using email and social media such as Twitter, Facebook, and WhatsApp. Digital flyers, social media posts, and emails included a REDCap survey hyperlink for prospective participants to register their interest and preferred details for follow-up discussion. After each interview, participants (both women and professionals) were asked to share the invitation to participate within their own networks or suggest other relevant people that might like to participate. These strategies aimed to reach a broad audience, whilst complying with stay-at-home directives in force at the time.

### 2.3. Data Collection

Semi structured interviews lasting 15–90 min were conducted with 17 women and 24 professionals, via Zoom video software or telephone. The average interview time was approximately 60 min. There were three shorter interviews with women that lasted approximately 15–20 min, due to the constraints of being at home alone with young children during the lockdown. These women shared openly with community researchers and rich data were obtained that made an important contribution to the findings reported in this paper. Interviews were audio recorded with consent. Four participants (two women and two professionals) did not provide consent for audio recording and detailed notes were taken in lieu. Community researchers [ST, MAA, ME, SH, AB] conducted interviews with women in their preferred language (see Table 2. Demographics of participating women). Interviews with health and social care professionals (see Table 3. Demographics of participating professionals) were conducted in English by researchers, including the lead author of this paper [FH]. This study obtained ethics approval from the Royal Children’s Hospital Human Research Ethics Committee (HREC/64046/RCHM-2020). Informed consent was obtained prior to each interview. Data from interviews with women were translated and transcribed by community researchers [ST, MAA, ME, SH, AB] and included reflective notes about the interpretation and translation process, to support data accuracy and integrity and assist with the analysis. Data from interviews with professionals were transcribed by professional services, then checked for accuracy and corrected as required by researchers [FH, ER].

### 2.4. Data Analysis

Data used in this reflexive thematic analysis [61] includes interview transcripts and reflective field notes written by the researchers and community researchers who conducted interviews [ST, MAA, ME, SH, AB]. Including the perspectives of professionals and women in the same analysis facilitated the integration of professional and system-level observations with women’s lived experiences and provided a rich overview of the perceptions and experiences of structural inequities during the COVID-19 pandemic. A constructivist worldview [58] and trauma and violence informed [52,53,59,60] approach informed researcher positionality throughout the analysis and all other stages of the research process. Reflexive thematic analysis is an interpretive method that emphasises the explicit acknowledgement, rather than elimination, of researcher positionality and bias [69,70]. Interpretation, a clear theoretical position, and high-level reflexive practices are used to reach new insights in the data—to produce high quality, rigorous, and trustworthy results [61,69,70]. Individual and team reflexive practices, including a trauma and violence informed approach, are discussed in further detail below, in the subsection ‘Reflexivity’. For this analysis, the lead author [FH] read each transcript multiple times to encourage immersion, referring to field notes where necessary for clarification and understanding. For interviews conducted in English, the audio recordings were also listened to. Transcripts were iteratively coded and categorised using NVIVO [71]. Themes were developed through reflexive supervision [FH, ER], team discussion, writing and re-writing [FH, SB, JS, ST, MAA, ME, SH, AB, ER], and handwritten visual maps. The lead author [FH] also documented handwritten and audio notes while reading, coding, and writing—to capture analytic thoughts and interpretations contemporaneously.

### 2.5. Reflexivity

This study was designed to include a constructivist worldview [58], as well as a trauma and violence informed approach—which acknowledges the existence and impact of structural violence as a source of trauma and re-traumatisation [52,53,59,60]. For example, a trauma and violence informed perspective facilitated reflexive acknowledgement of shared refugee experiences for women and the potential impacts of these experiences for women and the professionals providing their care. Our research team aimed to be sensitive, responsive, and accountable to the needs of participants at all times. Community researchers [ST, MAA, ME, SH, AB] shared language, cultural identity, and experiences with the women they interviewed. Researchers with healthcare backgrounds potentially shared professional identity and experiences with the professionals they interviewed [FH]. The lead author of this paper [FH] is a registered midwife with a clinical background, now working in public and maternal health research. Strategies to encourage self-enquiring reflexive practices were embedded from the outset. These strategies included regular individual supervision and post-interview debriefing; attendance at project meetings; group reflection; and ongoing iterative feedback processes to progress thinking and writing and to reach new insights [FH, SB, JS, ST, MAA, ME, SH, AB, ER]. Prior to commencing fieldwork, community researchers also attended tailored professional development with Foundation House, which focused on trauma-informed incidental counselling strategies and self-care, in the context of conducting fieldwork with research participants of refugee background [15,67].

## 3. Results

Interviews with 41 eligible participants were completed throughout August and September of 2020, including 17 women and 24 professionals. Participant demographic information is reported above in Table 2 and Table 3. To protect the anonymity of participants, whilst retaining context for readers, women’s responses have been labelled with their pregnant/postnatal status and self-described ethnicity. Professional roles have been de-identified and labelled as either community- or hospital-based. Potentially identifying interview quotes included in the results have been reported in the third person for the same reason. Results reported in this paper include three themes and four accompanying subthemes, as follows:

1. Structural inequities and the toll of the pandemic
  1.1. Staying at home—implications for wellbeing and safety in the Victorian context
  1.2. Amplified inequities
2. Supportive infrastructure
3. Cultural safety during the pandemic
  3.1. Accessing, understanding, and communicating health information
  3.2. Communication and cultural bridging—who, when, and why

### 3.1. Theme 1: Structural Inequities and the Toll of the Pandemic

Women and families of refugee background were inequitably impacted by the one-size-fits-all approach taken to implementing pandemic directives, including lockdown.

#### 3.1.1. Staying at Home—Implications for Wellbeing and Safety in the Victorian Context

Participants described implications for their wellbeing and safety during this time of extended isolation. As Melbourne grappled with a rise in COVID-19 cases and a second, stringent lockdown, other Australian States and Territories remained relatively open and COVID-19 free. With international travel limited and interstate travel to Victoria restricted, inviting visitors into your home not allowed and children attending school online [remote learning]—households were isolated.

*“…things had been good for the past couple of months and then suddenly things were getting bad again. It was cold, it was dark, everyone was getting sick.”*

(Professional, community-based)

Staying at home offered safety from contracting the virus, and the opportunity to spend more time together as a family. Although, as the lockdown progressed, negative social and emotional effects of living through a second extended lockdown arose. For some women, the pressure of the situation led to feelings of boredom, anxiety, and suffocation.

*“At the beginning of the restrictions, we were happy as it was the first time we were spending more time with each other as a family and spend more time with the kids, as they used to spend most of their time at school and we had just four hours with them daily before they go to bed, except the weekends and going away on holiday, but this situation took a very long time and it is so stressful, depressing, and feels like we cannot breathe anymore.”*

(Pregnant woman, Assyrian)

*“I feel throttled, sometimes, I feel upset, nervous, overthinking.”*

(Pregnant woman, Iraqi/Assyrian orthodox)

Many women were worried about being perceived as non-compliant with pandemic directives and were, therefore, reluctant to leave the house even for permitted reasons, such as exercise and grocery shopping. For these women, in the context of a refugee background, the visible presence of police in public areas contributed to fear and stress.

*“I feel scared when I go out […] so we just go [to the shops] around the corner. I am scared of the police. I would see the police car and would be scared.”*

(Postnatal woman, Karen)

While staying at home, many women who participated in this study were responsible for assisting their school-aged children with remote learning, despite not being able to speak, read, or write English themselves. The complexity of facilitating remote learning with other children at home and without an interpreter negatively influenced the way women felt about their pregnancies, or life with a new baby.

*“When I got pregnant, I was so upset. Because I got pregnant at a time like this. It was a lot of pressure on me and I was so depressed.”*

(Postnatal woman, Karen)

*“Online schooling is another thing that is affecting my pregnancy. I cannot talk to [my daughter’s] teachers unless there is an interpreter.”*

(Pregnant woman, Iraqi/Assyrian Orthodox)

*“Whenever am trying to help my older son, my toddler comes crying wanting something, or he fell down, and the baby as well [needs looking after]. To be honest it’s really hard, a lot has changed, I am exhausted.”*

(Postnatal woman, Sudanese)

Prioritising self-care and establishing new social connections was difficult. Staying at home with so much to do and limited support, it could be challenging for women to attend virtual appointments and online pregnancy or play groups.

*“… when the nurse visits me at home and sees what I have to deal with, and just the way she talks to me about how difficult my job is, makes me want to cry.”*

(Postnatal woman, Sudanese)

*“…women with young children need a positive atmosphere and to feel supported, especially if they don’t have anyone. I feel for us in particular you need someone to talk to remain emotionally sane, just to laugh and have fun.”*

(Postnatal woman, Sudanese)

*“… for a lot of mums with older children who are at home trying to homeschool, everything else, they just don’t have a lot of time to sit and [attend virtual groups, such as playgroup or group pregnancy care], for care and support…”*

(Professional, community-based)

While no women that participated in this study reported experiencing family violence during the pandemic, health and social care professionals (including those who worked with clients of refugee background) did perceive a general increase in family violence. Concerns were raised about the safety implications of not being able to physically see their clients, or their clients’ home environment, and whether telehealth was a safe or appropriate way to provide care in these situations.

*“…our cohort experiences family violence… I think when you then look at the documented high risk cohorts of like poor mental health, high stress, financial strain, unemployment, I mean the people seeking asylum fit into all of those categories so it makes sense that there’s a high rate in this population.”*

(Professional, community-based)

*“…the other thing that really escalated was the family violence situation […] there was a week when I was getting almost one referral each day. Yesterday was one such day when [the referrals] were back-to-back, two such situations.”*

(Professional, community-based)

*“…her [a client of refugee background] husband was so violent to her that she wasn’t even able to speak to me […] the restrictions made it easier for that to stay hidden.”*

(Professional, community-based)

*“I think that there are potential issues with family violence and […] women not being able to disclose, not being able to reach out to agencies to disclose family violence.”*

(Professional, hospital-based)

Professionals reflected that for families already in a healthy and stable environment, isolation could be a positive experience. Conversely, isolation had the potential to exacerbate pre-existing concerns for families experiencing challenges.

*“…some families have just loved the fact that they’ve been in a cocoon in that, in that isolation cocoon. But those are families that are in safe relationships, that have safe and secure incomes and housing. You know, they have, they’re in a positive space.”*

(Professional, hospital-based)

#### 3.1.2. Amplified Inequities

In some circumstances, the application of pandemic restrictions was viewed by professionals as fundamentally inequitable, with the potential to widen health disparities for people of refugee background—including an increased risk of COVID-19 exposure as well as poorer maternal and neonatal health outcomes. Existing barriers to quality care that were considered to be amplified by the pandemic restrictions included poverty, a lack of accessible transport, not being able to speak English fluently or at all, and the intersectional impact of such factors on health and system literacy.

*“Yes, so again, it’s always been people on the margins of society and poor people that have had access barriers and I guess, that’s all been magnified by the pandemic for 101 reasons. I mean, people who are impoverished, who are marginalised have an increased chance of getting COVID and an increased chance of complications of COVID and death, and have all of those pre-existing barriers of transport and language, and lack of data to do internet, calls. So, it’s all been magnified.”*

(Professional, community-based)

There were two circumstances demonstrating the inequitable application of pandemic restrictions for women and families of refugee background that were raised frequently by professionals during interviews. Restrictions on the presence of partners and support people during antenatal, birth, and postnatal care, and the rushed lockdown of a government-subsidised apartment complex, ‘The Towers’—which is described in the introduction. Professionals observed that in institutional environments such as hospitals, and when implementing large-scale policies and procedures, women and families who have the capacity to advocate for themselves are more likely to receive preferential treatment. Comparatively, people who are new to the Australian system and do not speak English have less power and are less likely to voice concerns or have their needs met.

*“And so a couple of things that happened were that one of the dads, the baby was born at, I think midnight or 11pm or something like that and then two hours later, the midwife said to him, ‘well it’s time for you to go home now’ […] this was a non-English speaking woman, obviously, you know, the father spoke a little bit of English, not a lot but, he was told at 2am, ‘that’s the rule, you’ve got to go home, you can come back tomorrow and stay for two hours’. So very obediently as most people who don’t speak the language take instructions […] he went home and came back the next morning at whatever, eight o’clock, nine o’clock, to see his wife and child. And as he walked in, there was a man who was in the room next door to them who, and they had also had their baby at, maybe, you know, midnight or one o’clock, and he walked out of the room and, you know, they’d talk to each other and congratulated each other because they met each other in the corridor and whatever. And so, the other dad who was Caucasian, spoke English, you know, local, said to him, ‘so why did you go home, I’ve been here all night?’.”*

(Professional, hospital-based)

*“…all the migrants that I spoke to, all of the refugees in ‘The Towers’, when you knocked on the door or when you rang them, they were really suspicious and they would only ease off when they knew that you were there to ask about their health concerns and nothing else. And nearly every one of them said ‘We’re generally ignored and now that suddenly we’re at risk of infecting the rest of the city, people are on our backs and there’s questions every day and once this is over, we’ll be forgotten again’. And it just rang so true because the economy was at stake and lockdown was at stake and all of that, there was suddenly all of this attention on a group of people who had been largely ignored.”*

(Professional, community-based)

*“Listen to communities […] ‘The Towers’ is a perfect example; they’re actually strong communities and there’s community leaders within there and there weren’t conversations with them about this. It could have been done differently.”*

(Professional, community-based)

Women and professionals expressed concern about the cultural and gendered implications of not having access to support. For example, by limiting the presence of support people, women received information and care alone, while fathers and support people missed out on critical education—including opportunities to learn how to be involved in caring for a baby. Some professionals also observed potential benefits to limiting the presence of support people. They felt that women who required an interpreter potentially experienced greater autonomy, compared to when English-speaking support people were present. For example, women may be less likely to be influenced by the opinion or presence of the support person and may have greater opportunity to voice their own preferences, questions, or concerns. However, this perceived benefit was not reported by any of the women who participated in this study.

*“Due to the restriction rules our family and friends are no longer can visit us at the hospital or at home, which also means we can’t celebrate the birth of our baby with family or friends. Family and friends visiting is an important thing in our culture after the birth of baby, and it’s an important event to celebrate with our friends or family. It’s a bit sad that I can’t share this happy time with others…”*

(Pregnant woman, Hazara)

*“I cannot take any support person with me, although I have a history of miscarriage before this pregnancy.”*

(Pregnant woman, Iraqi)

*“I do not have anyone, and the closest people could not come to me.”*

(Postnatal woman, Chaldean)

*“…her partner hasn’t been able to come and join her [in Australia] […] because he wasn’t a citizen, he couldn’t make it, so she had her baby by herself.”*

(Professional, hospital-based)

*“…when women are […] having all the education, having all the skills taught to them without their partners, is not going to be very beneficial for a homelife when the father can have a main role as well in caring for their child—because of this separation that’s taking place.”*

(Professional, hospital-based)

*“When women are alone at pregnancy appointments, they’re able to make their own choices.”*

(Professional, hospital-based)

Professionals who worked with women seeking asylum pointed out that this cohort was affected by pandemic restrictions to an even greater extent, due to the absence of Medicare and other social benefits. The inequitable application of restrictions and lack of financial assistance limited women’s ability to comply with healthcare recommendations, meaning that important care plans were unable to be enacted.

*“…she’s [client] seeking asylum so doesn’t have Medicare, and the hospital’s trying to get her to go out, external for a growth scan, because they didn’t want people in the hospital, and she didn’t have the money for that.”*

(Professional, community-based)

*“…someone who is an asylum seeker with a history of trauma, who has had the support of perinatal mental health through the pregnancy […] the recommendation is for an extended stay to provide that care and evaluation, and to see the necessary supports, for them to transition from hospital to home. But of course, that person can’t stay while her [other children] can’t come to the hospital [due to COVID-19 restrictions]. So of course, while the [healthcare] recommendations are in place, it’s just not feasible when these COVID-19 restrictions are in place with no flexibility.”*

(Professional, hospital-based)

### 3.2. Theme 2: Supportive Infrastructure

This theme describes financial, social, and faith-based infrastructure that provided support to women and families of refugee background during lockdown. This infrastructure is relevant to consider when contextualising access to maternity and early parenting care—given the relationship between women’s social, emotional, and financial circumstances, and maternal and neonatal health outcomes [11].

Most women that participated in this study experienced a significant change to their financial circumstances during the pandemic. Husbands, partners, and other family members (in multi-generational households) either lost their jobs or were working reduced hours, due to the effects of lockdown on businesses and employment. Some also chose to leave their jobs due to fear of contracting and transmitting COVID-19. Most women reported that at the time of these interviews, their households were receiving a form of government-funded pandemic payment as the main source of income.

*“…since the pandemic and quarantine time started my husband stopped working.”*

(Pregnant woman, Hazara)

*“Yes, his [participant’s husband] job has been reduced, but he is taking the JobKeeper [government-funded pandemic payment].”*

(Postnatal woman, Chaldean)

*“…she [participant’s mother who lives in the household] was scared to go to work [because of the pandemic] so she left work.”*

(Postnatal woman, Karen)

*“…the government helps us, like the current $550 [AUD] extra [government-funded pandemic payment] because of COVID.”*

(Postnatal woman, Sudanese)

Because of access to government-funded pandemic payments, most women felt their family’s income was adequate during lockdown. Pandemic payments provided vital assistance for families and potentially prevented experiences of financial stress and poverty, particularly while having a baby. For example, women who felt their family’s income was inadequate described having to make difficult choices, such as not being able to buy their children clothes in order to pay for rent or utilities.

*“…we have enough to live on…”*

(Pregnant woman, Karen)

*“…it [government-funded pandemic payments] was enough… just enough… for food… for family… sometimes.”*

(Postnatal woman, Karen)

*“…sometimes things like shopping for the children, there are some things you have to just cancel, so you can afford rent or electricity bill.”*

(Postnatal woman, Sudanese)

*“…the added pressure of, ‘how am I going to pay my rent, how am I going to buy the baby’s food’, takes away the joy. Also adds to a health concern.”*

(Professional, community-based)

Professionals who worked with people seeking asylum had clients without access to government-funded pandemic payments, who were living in severe poverty. Furthermore, restrictions to services supporting asylum seekers were also having negative and inequitable impacts on families in these circumstances, because less resources were available to people seeking asylum during the pandemic compared to pre-pandemic.

*“…people [seeking asylum] are just so destitute and it’s not going to get better.”*

(Professional, community-based)

*“…it seems more difficult for [women and families to receive material aid in a timely manner. Which is important […] that obviously contributes to anxiety for women […] they’re 40 weeks and they haven’t got any baby clothes or a bassinette or anything.”*

(Professional, community-based)

For women of refugee background, the ability to maintain strong community connections through social media, video software, and the telephone provided an informal infrastructure that supported social and emotional wellbeing. Family, friends, faith, and religion were an important source of reassurance and resilience during lockdown—along with personal practices such as prayer and reflection. Many women reflected that while their present circumstances were challenging, they felt much more concerned about the wellbeing of family and friends overseas.

*“…I depend on my religion. I take everything in prayer [when having troubling thoughts].”*

(Pregnant woman, Karen)

*“…we have a small community that is well connected, and some of my neighbours that I video chat with at the moment.”*

(Postnatal woman, Sudanese)

*“…we are relying on God to protect us.”*

(Pregnant woman, Assyrian)

*“Yeah, we are still meeting [for church, online] every week and seeing each other each week […] It is not the same as seeing each other face-to-face but at least we get to see each other.”*

(Pregnant woman, Karen)

*“Things are more difficult there [overseas], especially in the refugee camp. So, I feel like we don’t have many worries here, compared to there.”*

(Pregnant woman, Karen)

*“They’re really being impacted and they don’t complain […] people who’ve been through pretty full-on things in the past, and people that have, don’t take as much for granted, so there’s really incredible resilience as well.”*

(Professional, community-based)

### 3.3. Theme 3: Cultural Safety during the Pandemic

For women of refugee background, the extent to which women were able to access culturally safe health and social care influenced access to, and understanding of, health information. In practice, important elements of cultural safety included trust and relationship-based continuity of care, and consistent access to information and care in preferred language/s. Multicultural health workers, such as interpreters and bicultural workers (professionals who work with organisations and people that they share lived experience with, using their cultural knowledge, language skills, and community connections [72]), supported health and system literacy by facilitating culturally safe access to health information—meaning that women were able to understand pandemic directives and engage with their care in a meaningful way.

#### 3.3.1. Accessing, Understanding, and Communicating Health Information

Women of refugee background preferred to receive health information and information about the pandemic directly from trusted sources. These sources included family, friends, and community members; healthcare providers who could speak their language or with whom they had continuity—such as general practitioners [GPs], midwives, and maternal child health nurses; and community health and social care organisations that facilitated consistent access to interpreters and bicultural workers.

*“I have got family close by [who can communicate pandemic information to me] …”*

(Postnatal woman, Sudanese)

*“…within our community and among our friends, people share about it [pandemic information].”*

(Postnatal woman, Karen)

*“Mainly [I get health information from] my GP, who is from an Arabic background and speaks my language.”*

(Pregnant woman, Iraqi)

*“…the [maternal child health] nurse gave me contact numbers like [name of community-based refugee health and social care organisation] […] after birth I struggled with breastfeeding and the maternal child health nurse recommended I go to a place where I could get help with that…”*

(Pregnant woman, Assyrian)

*“I think I will ask my midwife [about any questions or concerns].”*

(Pregnant woman, Karen)

*“…the case manager from [community-based refugee health and social care organisation] called us and informed us with that [pandemic information]. They are calling us every time there is new information.”*

(Pregnant woman, Assyrian)

*“I feel like it would be nice to have a specific person to actually support mums and bubs during this time […] that we can call once or twice a week.”*

(Pregnant woman, Karen)

Some women felt confident about their understanding of health and pandemic information. Others were concerned they did not understand the Australian health system well and did not know where to go or who to call in an emergency, including if they had worries about their pregnancy or baby.

*“No, I do not know [where to go if something urgent happened].”*

(Pregnant woman, Iraqi)

*“I feel worried about my pregnancy because of the lockdown and I know that we can call an emergency line to ask in case of emergency, but I am not sure if that applies for pregnancy problems. I do not know the system well, or if they have a [telephone] line for pregnant women at the hospital to call in case of emergency.”*

(Pregnant woman, Iraqi)

Professionals perceived that women who were recently arrived and/or could not speak, read, or write English experienced inequitable access to information, due to gaps in the Australian Federal and Victorian Governments’ responses—which initially did not proactively engage with refugee and migrant communities or provide sufficient information in languages other than English.

*“…the refugee population from the family’s point of view, they come here, it’s a new country, they have very little English, they don’t know how to navigate the system, they don’t know—they’re not aware of the supports that they can access to start with.”*

(Professional, hospital-based)

*“This was evident when the pandemic resurged, when the infection rate resurged in certain groups of people and then, everyone suddenly wanted leaflets in Somali and leaflets in Karen and Amharic, which before didn’t exist. Then suddenly, the focus was on, “Oh, what can we do to increase literacy in migrant groups”. It’s too late, you know.”*

(Professional, community-based)

*“My concern is that this [pandemic response] works alright for the vast majority of women who are well educated, well literate, who speak English, who understand what has been told to them, who recognise when things aren’t going as normal as they would expect and so will access healthcare. Whereas there’s a whole group of women who are not in that situation, who don’t have the health literacy, who don’t have the language skills, who don’t have the ability to recognise what is normal and what is abnormal, and those are the women we might fail.”*

(Professional, hospital-based)

*“I guess it’s really more of a structural thing around engagement with the [Victorian Government] Department of Health […] what this has amplified is the lack of cultural conversations with cultural leaders and communities […] the work needs to be done to work with communities directly, and community leaders, so they can respond in ways that are effective…”*

(Professional, community-based)

Professionals who worked for community-based organisations, or for services that specialised in providing care for people of refugee background, felt it was necessary to communicate proactively and in new ways with their clients during this period of the pandemic. For example, by working closely with community members, community leaders, interpreters, and bicultural workers to develop culturally safe, trustworthy, and relevant communication strategies in community languages. These strategies involved both oral and written communication, including the use of video software and social media—to account for different levels of literacy, ability, and ways of communicating, such as strong oral traditions and storytelling.

*“…in the beginning it was quite challenging because every consult, every patient encounter would involve trying to word people up on what they can and can’t do. The fact that they could still consult with us, that it was okay if they travelled, if it was a face-to-face consultation. And then also try to navigate lots of mixed messages and we were all new to this and governments were new to this […] then used our cultural staff to educate patients and used other agencies, even the churches and other community centres…”*

(Professional, community-based)

*“I asked them [professionals] all to set up WhatsApp groups […] so that any of those COVID-19 updates, or any important information could be passed on very easily [to clients].”*

(Professional, community-based)

*“Basically, we’re ringing all our clients and explaining things over the phone, and finding out what resources they were accessing, what kind of information they were getting […] part of our role is finding out the best way to get information to clients…”*

(Professional, community-based)

*“[We] put up on Facebook with the [voice recorded] translation: ‘you are allowed to go out, you are allowed to do exercise, you can go out for a walk, five kilometres, come back’, anything that’s relating to mental health…”*

(Professional, community-based)

#### 3.3.2. Communication and Cultural Bridging—Who, When, and Why

Interpreters and bicultural workers bridged the gap between organisations and communities, increased access to care for families, and communicated culturally safe health information during a time of crisis. Continuity of care with interpreters and bicultural workers was identified as an important protective factor that improved the quality of communication, for both women and professionals. Women who had regular access to an interpreter and/or bicultural worker reflected more positively and confidently about their health and system literacy and experiences of care. Professionals who worked closely with interpreters and/or bicultural workers felt better equipped to respond well to women’s needs and concerns and were more confident that their organisation was meeting the cultural needs of community groups that engaged with the service.

*“I always have [the bicultural worker], so I gather all the information and then I say to them, ‘I’ll get [the bicultural worker] to ring you about that’, which has been great for me because […] if I didn’t have [the bicultural worker] I think that would be really hard to know how to reassure [women].”*

(Professional, community-based)

*“I think patients have found it harder to access care at mainstream services during the pandemic. Simple things like gatekeeper [COVID-19 screening] questions. So, they’d turn up and the gatekeeper would ask them questions about their exposure to COVID and if there’s no interpreter they can’t answer the questions, they can’t access the service. So, a lot of people have come to us because our gatekeeper either speaks their language already or will be able to very rapidly get a phone interpreter and ask those questions and let them in.”*

(Professional, community-based)

*“…our bicultural worker, she’s amazing. She does so much more for the women than she probably needs to do. The women are always calling her for, you know, outside hours and asking her for help with this, this and this.”*

(Professional, hospital-based)

*“And then [the clients] are all so desperate. So everything, every client will ring [the bicultural worker] up for every single reason they will face during COVID.”*

(Professional, community-based)

Women and professionals expressed that it was often harder to establish trust and rapport over the telephone and by telehealth, compared to face-to-face. This applied to all women receiving care from services, although was more pronounced for women who were not proficient in English. Without visual cues, it could be difficult to get a sense of how the appointment was going and to understand whether the woman was getting the information she needed.

*“I feel as though it is more comfortable if I see them [professionals] physically.”*

(Pregnant woman, Karen)

*“…it [telephone appointment] is better than nothing […] but the normal [in-person] one is better as we understand better.”*

(Pregnant woman, Assyrian)

*“I think with telehealth and interpreters, that’s been the major thing that I’ve noticed that just isn’t working.”*

(Professional, community-based)

*“So, losing face-to-face interpreters means that we’ve lost that whole personal connection. We can’t use the body language. I really can’t tell whether, because although I don’t understand the languages, you can tell by the body language that the patients and the interpreters are understanding each other […] I get really concerned about what’s being interpreted. I can’t use all those little cues.”*

(Professional, hospital-based)

*“It’s very challenging for the [client] and really challenging for the worker […] trying to get somebody connected to a phone call with a person who they’ve never met, talking about highly sensitive things.”*

(Professional, community-based)

*“…not being able to always request a [particular] gender interpreter for example. You just take who’s available. It’s not that you can’t do things as thoroughly on the phone, it’s just I think when using interpreters there’s so much that you interpret from the person’s face about understanding or clarification, there is something about that second, third-hand communication that being in person certainly makes things, certainly is better, I think.”*

(Professional, community-based)

Some professionals in hospital-based settings were concerned that women who needed an interpreter were receiving much less information than women who did not, as routine appointment times had been shortened to limit face-to-face contact. Across hospital and community-based settings, professionals with the autonomy to choose often continued to provide face-to-face appointments, as they felt this was essential to maintain a safe service. For example, the woman and interpreter may both attend the appointment in person, or the woman may attend the appointment in person and access an interpreter over the telephone. While mitigating the risk of COVID-19 transmission was appreciated and planned for, there was also an understanding of the bigger picture for women of refugee background and what it takes to effectively provide information and care. While women who participated in this study generally preferred face-to-face care, they did not voice the same concerns about receiving less information over the telephone.

*“I don’t know of any health service that actually makes appointments that you need an interpreter longer than those that don’t. Because you have the same whatever, 15, 10 min appointment with a person who speaks English, has no issues, as you have with a person who doesn’t speak English and may have a whole lot of issues that require an interpreter. It is obvious.”*

(Professional, hospital-based)

*“…there’s still always the option to come in as well, so if it’s somebody who really struggles with that, or some more vulnerable client they’re still coming in anyway.”*

(Professional, community-based)

*“…needing to get people in who need their blood pressure checked, or a lot of our clients don’t feel comfortable going to usual pathology ‘cause they don’t use interpreters, so we do quite a bit of the blood taking, especially the fasting ones first thing in the morning.”*

(Professional, community-based)

*“…within my unit, we’ve pretty much been able to keep our antenatal appointments [face-to-face] as required [due to providing specialist medical services]…”*

(Professional, hospital-based)

*“So, yes, we should be offering phone consults, but if we have someone who we think will miss out on very important things by offering a phone consult, then we have to offer face-to-face and yes, we can change things and adapt things, so we can make sure that people who are coming in are at low risk of having COVID.”*

(Professional, community-based)

## 4. Discussion

The results reported in this paper indicate that women and families of refugee background experienced inequitable access to health information and maternity and early parenting care during the extended second lockdown of the COVID-19 pandemic in Victoria, Australia, 2020. Factors that negatively impacted health equity for participants included limited access to health information, family separation and isolation, mental health concerns, and inadequate household income. Factors that positively impacted health equity for participants included access to social security payments, social connection to faith- and community-based networks, consistent access to bicultural workers and interpreters, and two-way community engagement between communities and health services.

### 4.1. Access to Health Information

Global morbidity and mortality related to COVID-19 has disproportionately affected racial and ethnic minority communities, including people of refugee background [47,48,50,73]. In Australia, public health directives have focused on increasing individual and organisational capacity for COVID-19-safe behaviours by sharing health information [26,74,75]. However, there is increasing evidence that the Australian and Victorian Governments’ initial pandemic response did not meet the health information needs of refugee background, migrant, and other communities (particularly those who do not speak or read English and/or who are not literate in their own language) and that this contributed to health inequities [50,75,76,77,78]. For example, during the second COVID-19 ‘wave’ in Victoria, there were more deaths among those born overseas than those born in Australia [79]. For communities of refugee background, good quality health information is accessible, accurate, culturally safe, and relatable [74,76,80,81]. Little is known about the specific health information needs of women of refugee background having a baby during the pandemic. Women who participated in this study preferred to receive information from a trusted source—meaning someone they knew, who could speak their language.

The results described in this paper indicate that inequitable access to health information affected the capacity of some women of refugee background to navigate the health care system, understand their rights, and advocate for themselves when receiving maternity and early parenting care during the pandemic. This finding is relevant to other research about effective communication of COVID-19 health information [74,76,80,81]. An Australian scoping review [80] identified factors that adversely influenced communication of ‘COVID-19 health information and engagement of ethnic minorities’ [80]. These include a lack of timely, good quality, or culturally safe health information; insufficient government engagement with communities; fear of authority; and low health literacy [80]. A qualitative study by Pourmarzi et al. [76] identified similar barriers for ‘migrants accessing official COVID-19 vaccine information’, including a lack of resources available in languages other than English, over-reliance on written rather than audiovisual resources, poor quality translation of written resources, and a one-way, top-down communication style. Many professionals who participated in Listening to What Matters were actively engaging with communities (including women of refugee background having a baby) in an attempt to address communication gaps by working closely with bicultural workers and interpreters. The results reported in this paper provide evidence that bicultural workers and interpreters made a critical contribution to culturally safe maternity and early parenting care [82], as well as to the pandemic health response. During the pandemic, bicultural workers and interpreters facilitated engagement between organisations and communities, shared rapidly changing information with women and families who had limited fluency in English or knowledge of the health care system, and facilitated two-way communication that built capacity and health literacy [74,76,80,81].

Community engagement is a strengths-based approach that has the potential to facilitate understanding of the needs, strengths, and challenges of a group through strategic consultation [74,76,81]. Community engagement can facilitate understanding of barriers and enablers to inform public health strategy, as well as building community trust in public health directives [74,76,81]. The World Health Organization recommended, early in the pandemic, that community engagement be prioritised locally and globally in the public health response [83]. Research from earlier pandemics and other large-scale disasters also identified early, meaningful, and ongoing community engagement as a key recommendation to facilitate the communication of good quality health information [80,81,84]. The application of hard lockdown provisions in ‘The Towers’ were seen by some participants in this study as a powerful example of the failure of public health measures to deliver information in a timely way, that overlooked existing leadership structures and communication networks already in place within communities [85,86]. This finding aligns with research by Mahimbo et al. [81], where Arabic-, Dinka-, Dari-, and Karen-speaking community members described community engagement as the ‘best way forward’, as this approach includes the opportunity to hear from community leaders, attend education sessions, ask questions, and raise concerns [81].

### 4.2. The Impact of Pandemic Directives on Structural Inequities

The findings reported in this paper indicate that some women of refugee background experienced cumulative negative impacts to their mental and social health during the pandemic that had the potential to amplify pre-existing structural inequities. For example, unequal access to health information, family separation and isolation, inadequate household income, and mental and social health concerns [48,75,87,88]. Many interviewees were concerned that appointment length was the same for telehealth, whether or not an interpreter was needed. Without the allocation of extra time to facilitate three-way communication, participants reported that women who needed an interpreter received less information and had less opportunity to ask questions or raise concerns. Family separation and isolation were pronounced due to the lockdown and disconnection from local and international family and community networks [25], meaning that women were unable to have culturally safe or expected support around them. For instance, help with meal preparation, housework, and childcare provided by friends, family, and community members. More extreme experiences of isolation shared by participants included birthing without a partner or support person present. While having more time at home together was a positive for some families [42], professionals had concerns that reliance on telehealth potentially had safety implications for women at risk of family violence, such as delayed disclosure, reiterating the importance of being able to intermittently visualise women and their home environment [89,90,91]. Government financial support was essential to most women who participated in this study, although still not enough to keep all participants receiving social security or pandemic payments out of poverty. Having a baby is a time when there are many necessities to be purchased, in addition to managing the cost of living—which can be a significant stressor for women [87,92].

When situating these findings in the context of women in Australia more generally, research increasingly depicts increased maternal anxiety, isolation, and unmet expectations of motherhood as common experiences during lockdown periods and when support people were excluded from attending care [35,42,43,44]. From a gender equity perspective, throughout the pandemic, women were more likely than men to experience poor mental health, family violence, and spend more time being the primary caregiver (including facilitating remote learning), as well as completing household duties like cooking and cleaning [87,88,89,92]. A recent cross-sectional analysis by Rees et al. [87] regarding the ‘prevalence of common mental disorders’ during the pandemic found that women of refugee background were at a greater risk of some mental health concerns than Australian-born women, due to fear and stress related to COVID-19 and material hardship during the pandemic [87]. It is evident from our findings that in addition to the above, women seeking asylum experienced heightened issues around service access and income, due to exclusion from some health, social, and economic services—which is also reflected in the national literature [48,50]. More research is needed to understand how the intersectional cumulative impacts of structural inequities have affected maternal and neonatal health outcomes (adversely or otherwise) for refugee background women during the pandemic; any differences in maternal and neonatal health outcomes between Australian-born and refugee background women and babies; the experiences of whole families (including multi-generational households); and the experiences of families living in rural and regional areas.

### 4.3. Recommendations

Recommendations for responding to future pandemics or other large-scale disasters that orient health equity for women of refugee background and align with the research used to support this discussion include the following:

1. Early and ongoing community engagement, including consultation with community leaders and trusted service providers.
2. The use of culturally safe communication strategies that leverage the existing strength, capacity, and leadership of communities.
3. Co-design of health information and dissemination strategies by and with community networks and leaders, as well as trusted service providers.
4. Presentation of health information in a variety of formats (particularly audiovisual).
5. Longer telehealth appointment length for women who require an interpreter and incorporation of face-to-face care at regular intervals.
6. Government and organisational investment in increasing diversity and representation in the health and social care workforce to improve cultural safety and experiences of care, including bicultural workers and interpreters.
7. Ensure social security payments are available to all women having a baby, to enable families to comply with public health directives; promote physical, mental, and social health; and prevent destitution—including those seeking asylum and/or on temporary visas.

## 5. Strengths and Limitations

An important strength of this research is the purposeful inclusion of women of refugee background (most of whom did not speak English). Community researchers recruited women through community networks and conducted interviews in preferred languages, which promoted cultural safety and trust. Interviews with women were translated and transcribed by community researchers, with reflexive notes taken alongside to assist with the data analysis, facilitating a high standard of data integrity. Interview data from women and professionals were rich and included unique insights into the experiences of women of refugee background throughout pregnancy and the postnatal period, during the pandemic. The geographic restriction for participant eligibility is a limitation of this research. Additionally, no fathers or other family members responded to the invitation to participate in this study. This meant the views of women from rural, regional, and other metro areas; fathers; and other family members were unable to be included.

## 6. Conclusions

The findings from Listening to What Matters provide new evidence regarding the experiences of women of refugee background accessing maternity and/or early parenting care in the north western suburbs of Melbourne, Australia. Cumulative negative impacts of pandemic-related public health directives had the potential to widen structural inequities experienced by women and families of refugee background who received pregnancy and/or early parenting care during the COVID-19 pandemic. For participants in this study, factors that improved access to health information and maternity and/or early parenting care included consistent access to health information in their preferred language, consistent access to an interpreter or bicultural worker, continuity of care with a trusted service or care provider, and access to social security payments. This paper also provides equity-oriented recommendations to inform future epidemics, pandemics, or other large-scale disasters.

## Figures and Tables

**Table 1:** Conditions/events experienced during the 2020 Victorian lockdown.

Pandemic Condition/Event	Explanation
Stay-at-home directive	Movement was allowed for ‘essential’ purposes only—meaning the activities and infrastructure required to maintain basic human safety and wellbeing. During ‘stage four’ of the lockdown, food, shopping, and exercise were permitted within a 5 km radius of home, masks were mandatory outside of home, and a nighttime curfew was in place [25,30].
Health care service change	Telephone and telehealth-based appointments were widely adopted to minimise face-to-face care encounters, wearing of masks and other personal protective equipment [PPE] became mandatory, screening questions were introduced at service entrances to limit the presence of people with COVID-19 symptoms and/or recent exposure, and the presence of partners and support people was restricted [25,31,32,33,34,35].
Remote learning	During the Victorian lockdown, most children attended school online, with exceptions primarily allowed for children of essential workers [25].
Employment	Operating restrictions placed on ‘non-essential’ services led to many people becoming unemployed or working less hours [25,36].
Lockdown of ‘The Towers’	From 4 July 2020, approximately 3000 residents living in the North Melbourne and Flemington public housing apartment complex were detained in their homes under a rapid ‘hard lockdown’—in response to an outbreak of positive COVID-19 cases associated with the complex. An investigation into the detention and treatment of residents in one of ‘The Towers’ by the Victorian Ombudsmen has since found the conditions enforced during this rapid lockdown violated their human rights, as people experienced inhumane conditions including going without food, medication, and/or other supports [37]. The Victorian Government later rejected the Ombudsman’s findings regarding the lawfulness of this response [38].
Supply chain disruption	The supply of some essential items was impacted by the pandemic, leading to shortages of essential items and retail stores placing temporary limits on the number of items able to be purchased in a single transaction [39].
Pandemic relief payments	To enable compliance with pandemic directives affecting employment and changes to income, the Australian and Victorian Governments introduced a number of pandemic-specific social security payments [19,25,40].

**Table 2:** Demographics of participating women.

Demographic Information (n = 17)	n (%)
Maternal age	
20–29	9 (52.9)
30–40	8 (47.1)
Children (excluding current pregnancy)	
1	3 (17.6)
2–3	13 (76.5)
≥4	1 (5.9)
Self-described ethnicity	
Karen	5 (29.4)
Sudanese	3 (17.6)
Dinka	1 (5.9)
Chaldean	2 (11.8)
Assyrian	1 (5.9)
Iraqi/Assyrian Orthodox	1 (5.9)
Iraqi	2 (11.8)
Jordanian	1 (5.9)
Hazara	1 (5.9)
Languages spoken ^a^	
English	7 (24.1)
Karen	5 (17.2)
Burmese	1 (3.4)
Arabic	8 (27.6)
Dinka	1 (3.4)
Assyrian	5 (17.2)
Chaldean	1 (3.4)
Dari	1 (3.4)
Country of origin	
Thailand	3 (17.6)
Myanmar/Burma	2 (11.8)
Sudan	3 (17.6)
South Sudan	1 (5.9)
Iraq	6 (35.2)
Jordan	1 (5.9)
Afghanistan	1 (5.9)
Years since arrival	
<1	1 (5.9)
1–4	9 (52.9)
≥10	7 (41.2)
Number of people living in household	
3	3 (17.6)
4–5	9 (52.9)
6–7	4 (23.5)
≥8	1 (5.9)
Pregnant/postnatal status at time of interview	
Pregnant	10 (58.8)
Postnatal	7 (41.2)

**^a^** Some women reported being able to speak more than one language.

**Table 3:** Demographics of participating professionals.

Demographic Information (n = 24)	n (%)
Professional role ^b^	
Obstetrician/General Practitioner/Paediatrician	4 (16.7)
Nurse/Midwife/Childbirth Educator/Doula	14 (58.3)
Bicultural Worker/Interpreter	3 (12.5)
Social Worker/Social Health Sector	3 (12.5)
Years in current role	
<1	2 (10.5)
1–2	5 (26.3)
3–4	4 (21.1)
5–9	3 (15.8)
10–19	3 (15.8)
≥20	2 (10.5)
Years in profession	
<1	1 (8.3)
1–2	2 (16.7)
3–4	1 (8.3)
5–9	1 (8.3)
10–19	4 (33.3)
≥20	3 (25)
Principle practice setting	
Hospital-based	10 (41.7)
Community-based	14 (58.3)

*Note.* Denominators vary due to missing values. ^b^ Professional roles have been grouped together to protect participant confidentiality.

## Data Availability

Data not available due to the conditions of ethical approval. Participants were assured that raw data would not be shared.
